# Gestational Diabetes and Future Risk of Diabetes

**DOI:** 10.4021/jocmr1201w

**Published:** 2013-02-25

**Authors:** Subash Chander Sivaraman, Sudheer Vinnamala, David Jenkins

**Affiliations:** a WISDEM Center, University hospital of Coventry and Warwickshire, Coventry, CV2 2DX, UK; b Birmingham Heartlands Hospital, Heart of England NHS Foundation trust, Birmingham, UK; c Worcestershire Royal Hospital, Worcester, WR5 1DD, UK

**Keywords:** Gestational diabetes, Glucose, Diabetes, Ante-partum, Post-partum

## Abstract

**Background:**

In this study of women with gestational diabetes we attempted to (a) Determine the magnitude of the long term risk of progression to diabetes and (b) Identify factors that predict the development of diabetes.

**Methods:**

All women diagnosed with gestational diabetes (GDM) at Worcestershire Royal Hospital, UK from 1995 to 2003 were included in this observational cohort study and followed up till 2009. Diabetes was diagnosed if fasting glucose ≥ 7.0 mmol/L, random/two-hour glucose following 75 gram oral glucose test (OGTT) ≥ 11.1 mmol/L or HbA1c ≥ 7.0%.

**Results:**

The risk of developing diabetes was 6.9% at five years and 21.1% at ten years following the initial diagnosis of GDM. Fasting and post-prandial glucose levels in the oral glucose tolerance test during pregnancy were associated with future risk of diabetes. There was no association with age, gestational age at diagnosis of GDM, numbers of previous and subsequent pregnancies.

**Conclusion:**

Risk of progression to diabetes in a UK based cohort of women with GDM is estimated. Women with fasting antenatal glucose ≥ 7.0 mmol/L and/or an antenatal two-hour glucose ≥ 11.1 mmol/L are at higher risk and need close follow up.

## Introduction

Pregnancy is known to cause a temporary state of insulin resistance that can unmask derangements in glucose homeostasis. Gestational diabetes mellitus (GDM) complicates 3-8% of pregnancies and is associated with adverse maternal and fetal outcome [1]. Delivery of the baby is usually accompanied by resolution of impaired glucose tolerance. GDM increases the chance of developing type 2 diabetes later in life [2].

The magnitude of the risk of post-partum diabetes varies depending on ethnicity, duration of follow up and criteria for diagnosis. The longest follow-up data suggest that up to 50% of women may develop diabetes over 20 - 30 years [3]. Various studies have attempted to identify subgroups of women who are more likely to become glucose intolerant later in life. The results have been inconsistent and sometimes contradictory [4]. Our objectives were a) to determine the long term risk of diabetes in a cohort of women with previous GDM, and b) identify which ante-partum and post-partum factors are associated with the size of the risk.

## Materials and Methods

This is a single centre, retrospective cohort study based at the Worcestershire Royal Hospital, Worcester, United Kingdom. All pregnant women attending the Worcester antenatal clinic considered at significant risk of GDM are investigated with a 75 g oral glucose tolerance test (OGTT), usually at 28 weeks gestation. Significant risk is identified if any one of the following factors is noted: first degree relative with diabetes, previous baby weighing 4.5 kg or more, pre pregnancy body mass index (BMI) ≥ 30 kg/m^2^ and South Asian/Middle-eastern/Afro-Caribbean ethnicity. GDM is defined as a fasting plasma glucose of ≥ 7.0 mmol/L and/or two-hour plasma glucose of ≥ 7.8 mmol/L. If GDM is diagnosed, the women are managed in a multidisciplinary diabetes antenatal clinic. Women with a previous history of GDM are not offered a repeat OGTT during a subsequent pregnancy, but are advised to monitor glucose levels from conception.

All women receive education about diet, exercise and self monitoring of glucose levels. If glucose levels are persistently more than 6.0 before meals, insulin treatment is commenced and titrated appropriately. All women are invited for an OGTT at eight weeks post partum. A significant number, however, fail to attend. Further screening tests for diabetes are at the discretion of the primary care physicians as there was no UK national guidance until 2008 [5].

Out of 481 women diagnosed with GDM over a period of 9 years from 1995 - 2003, 75 had no local record of post-natal glucose testing. The remaining 406 had their plasma glucose levels checked at least once after pregnancy. The numbers of women without diabetes but still having active follow-up dropped to 195 after five years, 108 after eight years and 66 after ten years.

We recorded the age, gestational age at diagnosis of GDM, fasting and two hour plasma glucose levels during antenatal OGTT. In addition we noted the numbers of previous and subsequent pregnancies for these women. The catchment population of this hospital during the study period was almost entirely Caucasian, however detailed ethnicity data was unavailable for our cohort. Women were included only once in the study even if they had GDM in subsequent pregnancies. We reviewed all investigations done as part of diabetes screening for these women until 31 July 2009. For the purpose of this study they were diagnosed with diabetes if any of the following were recorded: 1) Fasting plasma glucose ≥ 7.0 mmol/L; 2) two-hour plasma glucose ≥ 11.1 mmol/L; 3) random plasma glucose ≥ 11.1 mmol/l; 4) HbA1c ≥ 7.0% [6, 7].

The proportion of women developing diabetes at each time point was plotted on Kaplan-Meier curves and 95% confidence limits for diabetes incidence were derived. The effects of maternal age, gestational age (at diagnosis of GDM), fasting antenatal glucose level, two-hour antenatal glucose level and numbers of earlier and later pregnancies were assessed by fitting each factor in turn into a Cox proportional hazards regression model. Age, gestational age, fasting glucose and two-hour glucose were included in the model as linear covariates. Gestational age was missing for six women and two-hour glucose level was missing for one woman. These women, therefore, were omitted from the analysis to assess the effect of gestational age and two-hour glucose level respectively.

The effects of antenatal fasting and two-hour glucose levels were additionally assessed by comparing groups with fasting glucose levels in the ranges ≤6.0 mmol/L, from 6.1 mmol/L to 6.9 mmol/L and ≥7.0 mmol/L; and groups with two-hour glucose levels in the ranges less than 7.8, from 7.8 to 11.1 and greater or equal to 11.1.

## Results

**Figure 1:**
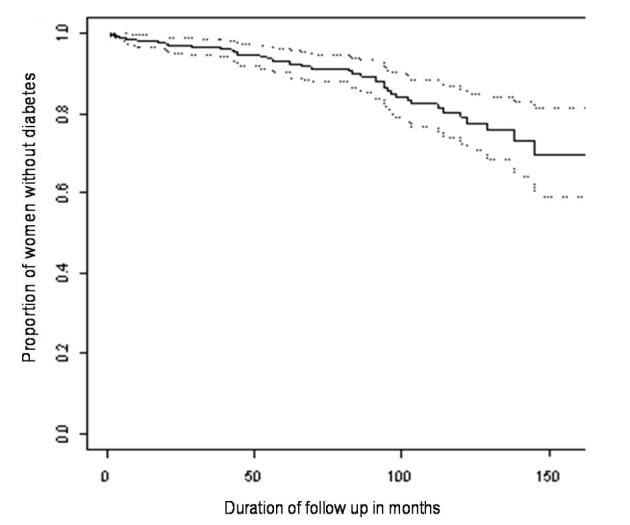
Kaplan-Meier estimates and 95% confidence interval for the proportion of women remaining diabetes free over time.

The median age of this cohort of women was 31 years (range 17 - 44 years). Prior to diagnosis of GDM 72 women had one previous pregnancy and 11 had two previous pregnancies. Following the index pregnancy 96 women had one subsequent pregnancy, 22 had two subsequent pregnancies and one woman had three subsequent pregnancies. At the end of follow up 43 women were diagnosed with diabetes. In five of these women the diagnosis was made solely on the basis of HbA1c ≥ 7.0% (range 7.3-8.7%). The Kaplan-Meier estimate and 95% confidence intervals of the proportion of women who remained free of diabetes following previous gestational diabetes is shown in Figure 1. At 60 and 120 months the estimates and 95% confidence intervals of the proportions of women with diabetes are 6.9% (3.8-9.9%) and 21.1% (14.1-27.5%) respectively.

The risk of diabetes increases significantly with the magnitude of the antenatal fasting glucose level (P < 0.0001) and two-hour glucose level (P < 0.0001). The differences between three fasting glucose level groups were highly statistically significant (P < 0.0001). The risk did not vary significantly with age at diagnosis of GDM (P = 0.4429), gestational age (P = 0.0756) or the number of earlier (P = 0.5312) or later (P = 0.7304) pregnancies.

Only one woman had a two-hour antenatal glucose level below 7.8 mmol/L. Women were divided into two groups depending on whether their antenatal two-hour glucose levels were less than or greater than or equal to 11.1 mmol/L The difference in risk of developing post-natal diabetes between these two groups was highly statistically significant (P < 0.0001). Compared to those with two-hour glucose less than 11.1 mmol/L, the risk of diabetes for women with a two-hour glucose greater than or equal to 11.1 mmol/L is increased by a factor of 9.6.

## Discussion

Our study confirmed that women in whom a previous pregnancy was complicated by gestational diabetes are at high risk of diabetes later in life. Studies done worldwide suggest that ethnicity is one of the key determinants of cumulative risk which varies from 70% in Sioux Indians, 13% in Chinese and 3.4% in Swedish Caucasians [8-10]. There are few data relating to women in the United Kingdom. We estimated the risk of post-partum diabetes to be 6.9% (3.8-9.9%) after 5 years and 21.1% (14.1-27.5%) after ten years.

Age is known to be a risk factor for the development of diabetes. In our population, however, the substantial risk of diabetes at ten years post-partum cannot be attributed to the age of the women as we did not find any association between the age at diagnosis of GDM and post-partum risk of diabetes.

Fasting and post prandial glucose levels from OGTTs during pregnancy have been analysed in many previous studies. Fasting glucose level was predictive in the majority of studies [11-14]. Most investigators adjusted for fasting glucose as a continuous variable. Hence a particular cut off value at which the risk increases could not be identified. Steinhart et al. found that a fasting glucose > 106 mg/dL (5.9 mmol/L) was associated with an 11-fold increased risk for future diabetes compared with fasting glucose levels < 106 mg/dL [15]. In our study we showed a significant 3.8 fold increase in the cumulative risk of diabetes if the fasting glucose was 6.1 - 6.9 mmol/L as compared to fasting glucose levels ≤ 6.0 mmol/L. When fasting glucose levels ≤ 6.0 mmol/L were compared with levels ≥ 7.0 mmol/L there was a 25 fold increase in risk of post-natal diabetes.

Previously published studies have used a variety of criteria to diagnose gestational diabetes, with glucose loads ranging from 50 grams to 100 grams [16, 17]. In our study in which the World Health Organisation criteria for diagnosis of GDM were employed, the two hour glucose level was predictive of future risk.

In a recently published study Ekelund et al noted a 4.8 fold increase in risk of diabetes if the antenatal HbA1c was ≥ 4.7% (28 mmol/mol). However the association with fasting glucose levels was more robust [18]. Some studies have concluded that use of insulin during pregnancy is a predictor of post partum diabetes while others did not find such an association [13, 19, 20]. We did not look for such an association in our cohort because the threshold to start insulin changed substantially during the study period.

A recent study in Danish women indicated that parity following a pregnancy complicated by GDM was associated with post-partum risk of diabetes depending on the age of the woman at the time of the index pregnancy [21]. Our study did not confirm this although we did not subdivide our study population by age.

The International Association of Diabetes and Pregnancy Study Groups (IADPSG) consensus panel has recently proposed new guidelines for diagnosis of GDM. Gestational diabetes is diagnosed if fasting glucose ≥ 5.1 mmol/L and/or two hour glucose ≥ 8.5 mmol/L following a 75 gram OGTT [22]. If these blood glucose thresholds are applied to our cohort, 133 women will be considered as not having GDM. Three of them were later diagnosed with diabetes. The IADPSG recommendations correlate closely with pregnancy outcomes, but it is still unknown whether the thresholds of fasting and two hour glucose levels accurately predict future development of diabetes.

Our study confirms that abnormal glucose homeostasis in pregnancy identifies a substantial risk of glucose intolerance later in life. A limitation of this study is its retrospective design. Information regarding other known risk factors for the development of diabetes such as family history of diabetes and pre-pregnant body mass index was available for only a minority of these women and was analysed.

### Conclusion

Studies worldwide have consistently shown that screening for diabetes in women with prior gestational diabetes is sporadic for a variety of reasons. Our study quantifies the risk in a UK based cohort for the first time. The risks at 6.9% after five years and 21.1% after ten years are clearly substantial. Women who had fasting levels ≥ 7.0 mmol/L or post prandial levels ≥ 11.1 mmol/L are at especially high risk and require close follow up. Current guidelines in the United Kingdom recommend annual fasting glucose measurements for women with a previous GDM. Further studies are required to refine this recommendation based on risk stratification.

## Bibliography

Presented as scientific paper in West Midlands Physicians Association Meeting at Birmingham on 12 may 2010.

## Abbreviations

GDM: Gestational diabetes mellitus
OGTT: Oral glucose tolerance test
mmol/L: Millimole per litre
